# Usefulness of Time-Point Serum Cortisol and ACTH Measurements for the Adjustment of Glucocorticoid Replacement in Adrenal Insufficiency

**DOI:** 10.1371/journal.pone.0135975

**Published:** 2015-08-28

**Authors:** Elise Rousseau, Michael Joubert, Géraldine Trzepla, Jean Jacques Parienti, Thomas Freret, Marie Christine Vanthygem, Rachel Desailloud, Hervé Lefebvre, Antoine Coquerel, Yves Reznik, PHAD Study Group

**Affiliations:** 1 Department of Endocrinology, Caen University Hospital, 14033, Caen, France; 2 Department of Clinical Research, Caen University Hospital, 14033, Caen, France; 3 UFR Pharmaceutical Sciences, EA4259, University of Caen Basse-Normandie, 14032, Caen, France; 4 Department of Endocrinology, Lille University Hospital, 59037, Lille, France; 5 Department of Endocrinology, Amiens University Hospital, Hopital SUD, 80054, Amiens, France; 6 Department of Endocrinology and INSERM U413, IFRMP23, Rouen University Hospital, 76031, Rouen, France; 7 Department of Pharmacology, Caen University Hospital, 14033, Caen, France; 8 University of Caen Basse-Normandie, Medical School, Caen, F-14032, France; Medical University Innsbruck, AUSTRIA

## Abstract

**Background:**

Adjustment of daily hydrocortisone dose on clinical criteria lacks sensitivity for fine tuning. Long term hydrocortisone (HC) over-replacement may lead to increased morbidity and mortality in patients with adrenal insufficiency (AI). Biochemical criteria may help detecting over- or under-replacement but have been poorly evaluated.

**Methods:**

Multicenter, institutional, pharmacokinetic study on ACTH and cortisol plasma profiles during HC replacement in 27 AI patients compared to 29 matched controls. All AI patients were administered HC thrice daily at doses of 6, 10 and 14 mg/m^2^/d. Blood samples were drawn hourly from 0800h to 1900h. The main outcome measures were: i) plasma peak cortisol and cortisol area under the curve (AUC) in AI patients compared to controls, ii) correlations between cortisol AUC vs single-point cortisol or ACTH decrease from baseline (ΔACTH) and iii) the predictive value of the two latters for obtaining AI patients’ cortisol AUC in the control range.

**Results:**

Cortisol peaks were observed 1h after each HC intake and a dose response was demonstrated for cortisol peak and cortisol AUC. The comparison of AI patients’ cortisol AUC to controls showed that 81.5% AI patients receiving 6mg/m^2^/d were adequately replaced, whereas most patients receiving higher doses were over-replaced. The correlation coefficient between 1000h/1400h cortisol concentrations and 0800-1900h cortisol AUC were 0.93/0.88 respectively, whereas the 0800-1200h ΔACTH fairly correlated with 0800-1900h cortisol AUC (R = 0.57). ROC curve analysis indicated that the 1000h and 1400h cortisol concentrations best predicted over-replacement.

**Conclusions:**

Patients receiving a 6mg/m^2^ hydrocortisone daily dose exhibited the most physiological daytime cortisol profile. Single point plasma cortisol correlated with daytime cortisol AUC in AI patients. Although hydrocortisone dose should be currently determined on clinical grounds, our data suggest that single point plasma cortisol may be an adjunct for further hydrocortisone dose adjustment in AI patients.

## Introduction

Glucocorticoid replacement in patients with adrenal insufficiency (AI) is based on the use of hydrocortisone (HC), but the appropriate dose and the timing of its administration remain controversial [1–3]. The rate of endogenous cortisol production has been accurately determined in humans to be in the range of 6 to 11mg/m^2^/d [4–6]. These findings have led some authors to recommend decreasing the HC daily dose to approximately 20 mg per day, optimally divided into three times per day [7,8]. The absence of evidence-based guidelines and objective criteria for treatment adjustment explains the wide range of recommendations for the optimal HC regimen. The physician’s judgment relies on clinical criteria which will detect only the extremes of under- or over-replacement [1]. Monitoring HC replacement with biochemical criteria, including urinary, salivary and plasma cortisol measurements has been proposed by some authors [8–12]. Urinary cortisol excretion is influenced by the daily dose distribution and the limited binding capacity of circulating cortisol-binding globulin. These pitfalls limit the contributive value of this biochemical marker for HC dose adjustment [9]. A few studies have proposed to measure plasma cortisol [8–11] or ACTH concentrations [13–16] after glucocorticoid oral intake for evaluating the quality of HC replacement. A recent pharmacokinetic study based on the plasma cortisol profile during HC replacement has emphasized that over- or under-replacement were frequent findings in patients replaced by a daily dose of HC not exceeding 20 mg per day and split in two or three daily intakes [17]. Nevertheless plasma cortisol measurement is not widely accepted for the monitoring of HC replacement and may be restricted to the detection of HC under-replacement [18]. The lack of accurate adjustment of HC replacement therapy may partly explain the two-fold mortality increase in AI patients compared to the general population [19,20]. Other possible adverse consequences of unadjusted HC replacement in AI patients include altered quality of life [21] and reduced bone mass density [22–24]. These difficulties reinforce the need to develop a valuable biochemical tool for HC adjustment. In this context, our aim was to evaluate the contributive value of time point cortisol and ACTH measurements for adjusting HC replacement in patients with AI.

## Subjects and Methods

### Subjects

This study was conducted in a group of 27 primary AI patients and a group of 29 healthy subjects recruited at the University Hospitals of Caen, Lille, Amiens and Rouen (France). The study was approved by the Caen University local Research Ethics Committee, and all participants gave their written informed consent. All patients had primary AI assessed by 0800h serum cortisol levels <50nmol/L and ACTH levels >13pmol/L in the absence of HC replacement. Table 1 describes the clinical characteristics of the AI patients. The subjects had no associated acute or chronic disease, had not been previously exposed to endogenous or exogenous glucocorticoid compounds other than HC and were not presently receiving either contraceptive estroprogestative or anti-epileptic compounds. Pregnancy was an exclusion criterion in premenopausal women. Chronic alcohol abuse was also an exclusion criterion. The patients’ renal, liver and thyroid biochemical measurements had to be in the normal range. Healthy subjects constituing the control group were matched to AI patients for age, gender and BMI. They had no associated acute or chronic disease, had normal sleep patterns and were not taking drugs that interfere with the hypothalamic-pituitary-adrenal axis.

**Table 1:** Clinical characteristics of the AI patients.

Subjects	Age	Sex	BMI (kg/m^2^)	Etiology of AI	HC dose (mg/d)	Fludrocortisone dose (μg/d)
1	58	f	20.5	Addison's disease	20	62.5
2	52	f	20.3	Bilateral adrenalectomy	20	50
3	42	f	24.8	APS II	30	100
4	54	m	27.7	Addison's disease	30	50
5	54	f	21.3	APS II	20	50
6	60	m	47.5	Adrenoleukodystrophy	30	
7	38	f	35	Addison's disease	20	150
8	35	m	23.7	Addison's disease	20	50
9	45	f	24.5	APS I		
10	36	f	20.5	APS II	25	50
11	49	f	18.7	APS II	30	100
12	58	f	31	Bilateral adrenalectomy	30	50
13	26	f	32.6	APS II	30	50
14	43	f	19.4	APS II	10	50
15	70	f	26.9	Mitotane induced AI	30	50
16	56	f	24	APS II	20	50
17	42	f	27.9	APS I	20	100
18	68	f	28.3	Addison's disease	30	75
19	41	m	25	Bilateral adrenalectomy	30	25
20	73	f	34.7	APS II	30	50
21	40	m	24.8	APS II	20	50
22	61	f	27.6	APS I	20	100
23	60	f	21.5	APS II	25	50
24	37	m	22.9	Addison's disease	30	150
25	20	m	23.7	Addison's disease	30	100
26	56	m	28	Addison's disease	25	50
27	50	f	21.5	APS II	15	50
**Mean ± SD**	**49± 13**	**19F/8M**	**26.1 ± 6.2**		**23.7 ± 7.4**	**63.4 ± 36.2**

AI, adrenal insufficiency; APS, autoimmune polyglandular syndrome; HC, hydrocortisone.

### Methods

The experiments were conducted at the endocrine unit or research unit of the study centers. On each day of the study period, patients were given HC replacement under fasting conditions thrice per day at 0805h, 1205h and 1605h (50%, 25% and 25% of the total daily dose, respectively). Each patient was given a dose of 6, 10 and 14mg/m^2^/day HC on three different days in a random order. Ten milligrams hydrocortisone tablets were used and quarter tablets were prepared in order to adjust the dose for each experiment. On each study day, peripheral venous blood samples were drawn hourly (± 5min) from 0800h to 1900h for cortisol and ACTH measurements. The evening and night periods were not studied considering the short half-life of HC and the negligible residual concentration of HC metabolites during these periods. The same sampling protocol was performed in the healthy control subjects for daytime cortisol measurement.

### Assays

Serum was separated from the blood samples immediately after collection and stored at -20°C until use. Cortisol concentrations were measured using a radioimmunoassay GammaCoat^TM^ kit [^125^I] (DiaSorin CA 1529–49, France). The intra- and inter-assay coefficients of variation (CV) were less than 7%. ACTH concentrations were measured using an ACTH ELISA assay (Cisbio, France). The lowest limit of detection was 0.22pmol/L. The intra- and inter-assay CV were less than 10%, except for the lowest range of the standard curve (inter-assay CV = 22%).

### Data analysis

For each experiment performed in AI patients and in healthy controls, the cortisol area under the curve (AUC) was measured at the following time periods: 0800-1200h, 1200-1600h, 1600-1900h and 0800-1900h. The intra-individual CV of the cortisol AUC determination assessed in AI patients receiving a 10mg/m^2^ HC dose on three different occasions was 9.6% demonstrating good reproducibility. The data analysis included several steps: first, cortisol peak amplitude and timing and ACTH concentrations were assessed at the different HC daily doses and compared by repeated analysis of variance (ANOVA). The normal physiological range of the cortisol AUC was defined as the mean cortisol AUC ± 2 SD among healthy controls, and the comparison of the patient’s cortisol AUC at each HC dose to the cortisol AUC range from healthy controls allowed to classify AI patient’s as adequately, under- or over-replaced. Thereafter, linear correlations were assessed between cortisol AUC and (i) single-point cortisol, (ii) single point ACTH (iii) the percentage of ACTH decrease from baseline at different time periods (ΔACTH, calculated as 0800h ACTH—nadir ACTH / 0800h ACTH). In case of overall significant effect of the HC dose, pairwise comparisons were performed by Turkey's tests. Finally, the variables that demonstrated the highest correlation with cortisol AUC were qualified for the determination of their sensitivity and specificity to predict the quality of HC replacement by the receiver operating characteristic (ROC) curve analysis. Quantitative variables were expressed as the mean ± 2 SD, and qualitative variables were expressed as percentages. According to HC dose and time, an F value higher than 3.84 qualified a statistical difference between the measured values for the comparison of plasma cortisol, cortisol AUC and ACTH levels. Pearson coefficients and the coefficient of correlation were computed for each correlation (R). Overall correlations were determined using appropriate methods that took into account the crossover design. Different thresholds and their corresponding sensitivity and specificity for predicting adequate or over-replacement were plotted on a ROC curve. The SAS and SPSS software packages were used to perform the statistical analyses. The time-point and AUCs for cortisol and ACTH concentrations of AI patients and healthy subjects are available in the S1 Supplement.

## Results

### Pharmacokinetic studies

**Fig 1:**
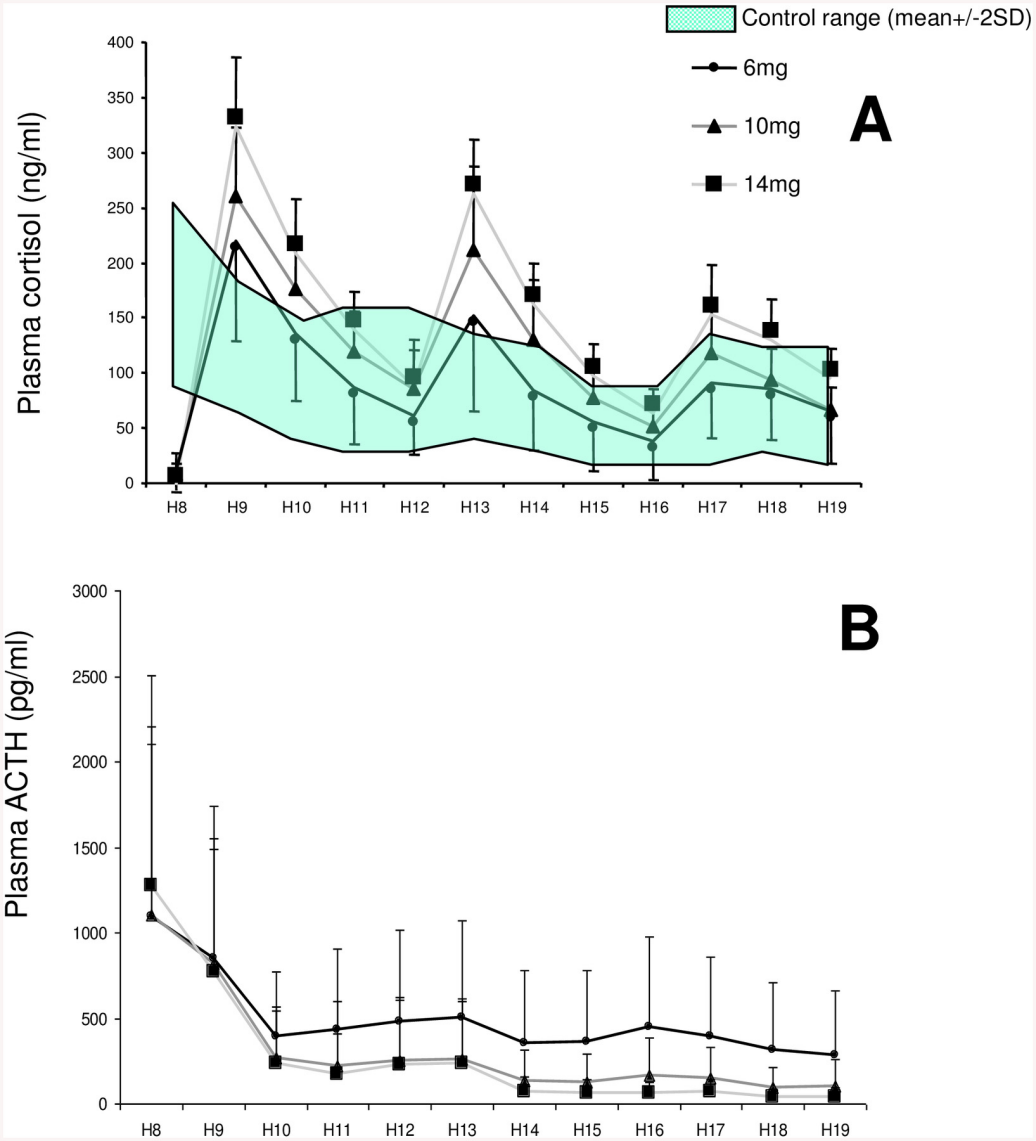
Mean daytime plasma cortisol (A) and ACTH concentrations (B) with hydrocortisone replacement (three times per day; 6, 10 or 14mg/m^2^/day). Normal cortisol range in healthy control subjects is represented by the grey area (mean ± 2 SD).

The mean daytime plasma cortisol concentrations from AI patients at the different HC replacement doses are depicted in Fig 1, panel A (data file in S1 Supplement). Considering all cortisol concentrations sampled every hour from 0800h to 1900h in HC treated AI patients, a significant difference in the cortisol level was observed compared to control patients, whatever the HC replacement dose [F(1,970) = 284.6, p<0.001]. When comparing the overall daytime cortisol levels measured after each of the three HC replacement doses—ie 6, 10 and 14 mg/m^2^—successively administered to each patient, a significant difference was observed between cortisol profiles at the different HC doses [F(1,970) = 67.7, p<0.001]. Three cortisol peaks were observed 1h after each HC intake—*i*.*e*. at 0900h, 1300h and 1700h, whatever the HC replacement dose considered. Furthermore, the magnitude of each peak was dependent on the HC replacement dose administered [For the first peak: F(2,403) = 20.7, p<0.001; for the second peak: F(2,403) = 22.5, p<0.001; for the third peak: F(2,403 = 23, p<0.001].

Individual plasma ACTH levels measured before HC intake varied over a wide range (data not shown). After the first HC intake at 0805h in the morning, the mean ACTH concentration sharply decreased whatever the HC replacement dose considered (Fig 1, panel B). Indeed, a significant decrease in ACTH level was observed from 0800h until 1400h [F(2,567) = 108.3, p<0.001], whereas no significant change was observed thereafter, *i*.*e*. between 1400h and 1900h [F(2,486) = 1.4, p = 0.24], demonstrating thus a nadir of ACTH plasma level occurring about 6h after the first HC dose intake. Besides, a significant difference was observed between ACTH profiles obtained with the three different HC replacement doses [F(2,970) = 13.74, p<0.001]. When comparing the overall daytime ACTH, a weaker ACTH decrease was observed at 6mg/m^2^/day compared to 10 and 14mg/m^2^/d HC doses (-72% *vs* -88% and -93% respectively, p<0.05).

The mean cortisol AUCs calculated in AI patients taking the three different HC replacement doses and in control subjects are shown in Table 2. On a 6mg/m^2^/d HC replacement dose, the cortisol AUC was in a close range from cortisol AUC of healthy controls. Thus, no significant difference was observed compared to healthy controls whether considering cortisol AUC for the overall sampling period (AUC_0800–1900h_), or the cortisol AUCs calculated over the time periods of 1200–1600h and 1600–1900h. In contrast, on the 10 and 14 mg/m^2^/d HC replacement doses, cortisol AUCs were significantly higher than cortisol AUC from healthy controls in the overall sampling period (0800h-1900h), as well as in almost all time periods (Table 2). Moreover, a generalized linear model for repeated data demonstrated a dose-response relationship for each cortisol AUC sampling period calculated after the three daily HC intakes, as well as for the cortisol AUC calculated for the overall sampling period [F(2,79) = 58.09, p<0.0001; F(2,79) = 76.98, p<0.0001; F(2,79) = 66.15, p<0.0001; F(2,79) = 81.75, p<0.0001, for respectively cortisol AUC_0800–1200h_, AUC_1200–1600h_, AUC_1600–1900h_, AUC_0800–1900h_].

**Table 2:** The cortisol AUC expressed as mean ± 2 SD (ng/mL/time period) and range values in normal healthy subjects and AI patients.

	AUC_0800–1200h_	AUC_1200–1600h_	AUC_1600–1900h_	AUC_0800–1900h_
**Control Group**	445±181	335±128	205±122	985±326
**(range)**	(264–626)	(207–463)	(83–327)	(658–1310)
**AI: HC 6 mg/m** ^**2**^ **/d**	558±284^1^	320±184	211±134	1089±506^1^
**AI: HC 10 mg/m** ^**2**^ **/d**	729±293^2^	487±344^2^	271±150	1488±654^2^

All statistics are expressed in comparison with control group:

^1^p<0.05,

^2^p<0.001.

AUC: area under curve. AI: adrenal insufficient patients. HC: hydrocortisone.

### HC dose adjustment based on plasma cortisol profiles

The individual comparison of cortisol AUC_0800–1900h_ from each AI patient on the three HC replacement doses to the normal range of cortisol AUC_0800–1900h_ defined in healthy controls showed that 81.5% of patients receiving 6 mg/m^2^/d HC were adequately replaced. In contrast, when receiving the 10 and 14mg/m^2^/d HC doses, most patients were over-replaced (81.5% and 96.3%, respectively). Whatever the HC dose administered, no patient was under-replaced (Table 3).

**Table 3:** Quality of HC replacement dose adjustment in AI patients who underwent three different HC regimens (6, 10 and 14 mg/ m2/d).

HC replacement dose (mg/m^2^/d)	6	10	14
Under-replaced n (%)	0	0	0
Adequately replaced n (%)	22 (81.5)	5 (18.5)	1 (3.7)
Over-replaced n (%)	5 (18.5)	22 (81.5)	26 (96.3)

AI: adrenal insufficient. HC: hydrocortisone.

The correlation coefficients between the cortisol AUC calculated over the different time-periods and either cortisol concentrations measured at different time-points, or ΔACTH concentrations, are shown in Table 4. The strongest correlations with cortisol AUC_0800–1900h_ were observed for cortisol concentrations measured at 1000h and 1400h (R = 0.93 and 0.88, respectively), while the cortisol concentrations measured at 1000h, 1400h and 1800h were respectively highly correlated with the cortisol AUC calculated for 0800–1200h, 1200–1600h and 1600–1900h periods. Considering ACTH measurement, the ΔACTH AUC_0800–1200h_ best correlated with cortisol AUC_0800–1900h_ although such correlation was weakest than all those calculated for time points cortisol concentrations (Table 4). In contrast, no significant correlation was observed between cortisol AUCs and time-point ACTH concentrations (data not shown).

**Table 4:** Correlation coefficients (R) between single-point cortisol and Δ ACTH plasma concentrations *vs* time-period cortisol AUCs.

		AUC_0800–1900_	AUC_0800–1200_	AUC_1200–1600_	AUC_1600–1900_
Cortisol concentration	1000h	0.93	0.95	-	-
	1100h	0.87	0.85	-	-
	1200h	0.74	0.71	-	-
	1300h	0.57	-	0.57	-
	1400h	0.88	-	0.92	-
	1500h	0.81	-	0.89	-
	1600h	0.78	-	0.88	-
	1700h	0.71	-	-	0.89
	1800h	0.83	-	-	0.94
	1900h	0.73	-	-	0.75
ΔACTH	0800–1000h	0.49			
	0800–1200h	0.57			
	0800–1400h	0.56			
	0800–1600h	0.55			
	0800–1900h				

AUC: area under curve.

**Fig 2:**
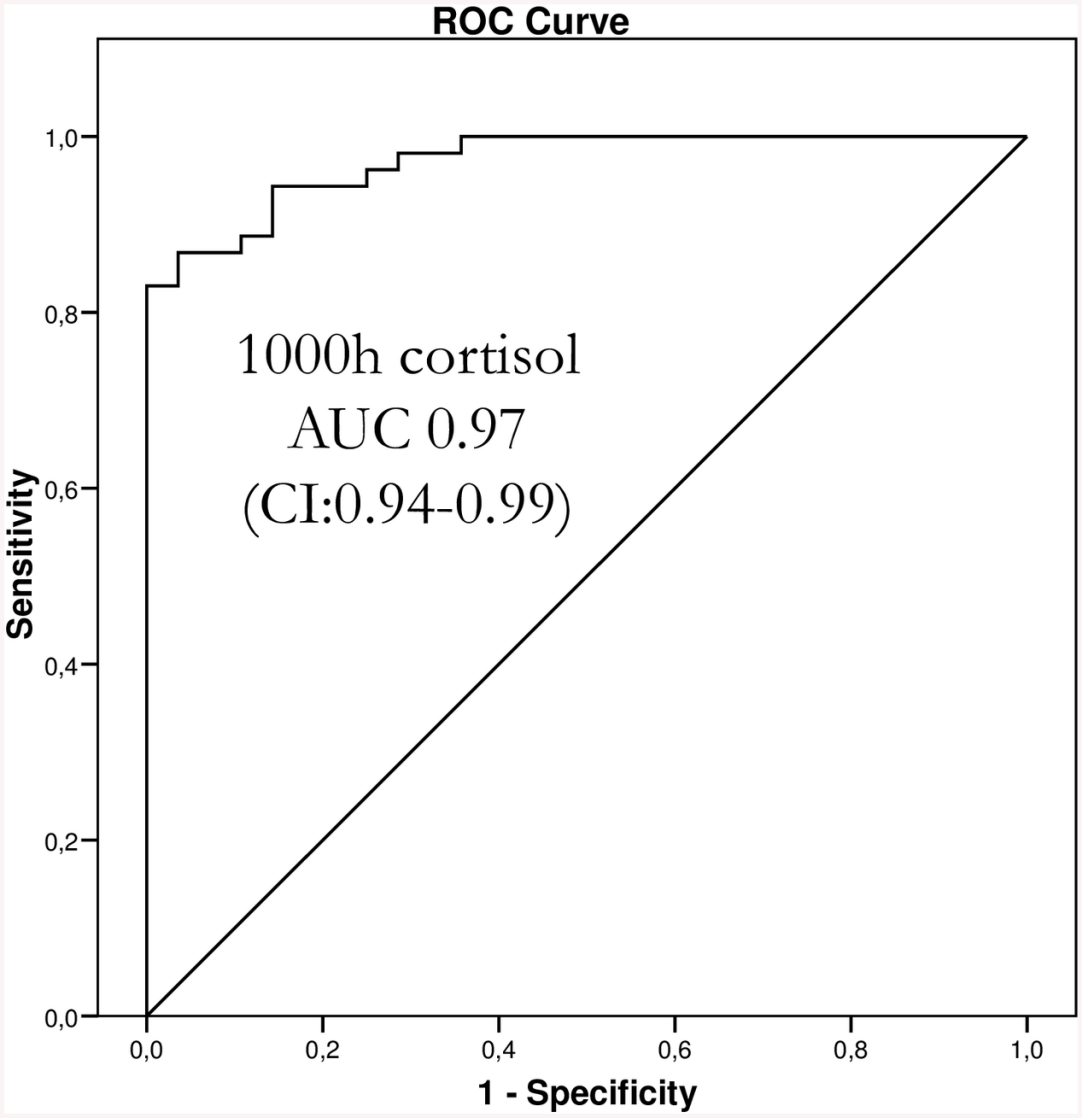
ROC curve analysis of the predictive value of single-point 10 am plasma cortisol for adequate or over-replacement as determined by the comparison of pooled AI patients’ cortisol AUC_8–19h_ to pooled healthy subjects’ cortisol AUC_8–19h_.

ROC curve analysis showed that 1000h cortisol level best predicted over-HC replacement (AUC 0.97, CI: 0.94–0.99, 95% sensitivity, 86% specificity) at a cut-off cortisol level of 402nmol/L (Fig 2), whilst 1400h cortisol level higher than 253nmol/L also fairly predicted over-replacement (AUC of 0.95, CI: 0.91–0.99, 92% sensitivity, 86% specificity). The predictive value of 0800–1200h **Δ**ACTH for HC over-replacement was less accurate (AUC 0.88, CI: 0.81–0.95, 83% sensitivity, 79% specificity) at a 0800–1200h **Δ**ACTH cut-off of -86%. All other cortisol time points and **Δ**ACTH variables were less accurate for predicting over-replacement (data not shown).

## Discussion

Owing to the absence of evidence-based guidelines for HC replacement, physicians adapt, so far, empirically doses and timings of administration based only on clinical evaluations. This leads to a large number of patients with non-optimal replacement therapy, thus affecting quality of life and increasing morbidity and mortality risks. Herein, we demonstrate that a single point cortisol determination (notably, at 1000h) seems to be an acceptable surrogate of the daytime cortisol AUC, which reflects glucocorticoid exposure during HC replacement period. Besides, we show that under a thrice daily HC replacement regimen, both doses of 10 and 14 mg/m^2^/d lead to over-replacement in most patients.

A major goal of the present study was to assess whether a single cortisol and/or ACTH plasma measurement could represent a valuable biochemical marker of adequate HC adjustment. Prior studies aimed to evaluate the helpfulness of plasma cortisol profile for a fine tuning of HC replacement therapy in AI patients. Based on the measurement of one morning and one evening time-point plasma cortisol, Peacey *et al*. have found that a majority of patients receiving a mean dose of 30mg HC per day were over-replaced and instead have recommended a 20mg mean dose splitted into three doses per day [10]. Similarly, Howlett established a score of replacement quality, based on three daytime plasma cortisol levels and a measurement of 24h free urinary cortisol. Based on this score, Howlett concluded that 20mg was an appropriate starting dose for HC replacement, and that a thrice daily regimen was the most appropriate regimen [8]. Mah *et al*. have conducted a pharmacokinetic study, which again validated a thrice daily regimen and have suggested that the total daily dose should be adjusted to the patient’s weight for reducing the inter-individual variability of the plasma cortisol profile [11]. Of note, most of these studies used, if any, reference values from published data or an arbitrary concentration range based on 2 to 3 time points of cortisol measurements [7,8,10]. A recent pharmacokinetic study improved the robustness of their reference values based on the measurement of 3 time-point cortisol concentrations a day in a sample of 60 control subjects [17]. These authors observed that 33% to 65% of patients on 20mg HC per day administered in a thrice daily regimen remained mostly over-treated, whilst about 10% were undertreated [17]. Our results are in accordance with litterature data, showing that HC doses commonly prescribed to AI patients (10 and 14mg/m^2^/d) are associated with over-replacement and that all the contrary, a daily HC dose between 6 and 10 mg/m^2^/d allows to obtain a physiological cortisol AUC in most patients. A strength of our study is the determination of the plasma cortisol AUC in HC-treated AI patients in comparison to a reference group of healthy subjects for the adjustment of HC replacement. Such criterion integrates the daytime cortisol profile which had not been considered in previous studies. Furthermore, our pharmacokinetic study demonstrates that cortisol and ACTH plasma concentrations both vary over time, with a mean cortisol peak occurring 1h after each HC intake, and an ACTH nadir occurring 6h after the first HC intake. Furthermore, a dose response relationship was observed for the three cortisol peaks, for the daytime cortisol AUCs and for the ACTH decrease after HC replacement.

In order to evaluate the relevance of cortisol and ACTH plasma measurements as biochemical tools to refine HC replacement therapy in AI patients, we examined the strength of the correlation between time-point cortisol/ACTH values and the daytime cortisol AUC. A strong and highly significant correlation was observed between the cortisol AUC calculated for the overall daytime period and the cortisol concentrations, at both 1000h and 1400h time-points. Mah *et al*. have also observed such a correlation between the cortisol AUC and the plasma cortisol concentration, both measured four hours after the time of HC intake [11]. However, the study conditions were restricted to a single and fixed 10mg morning dose of HC. Our results extend these findings and show for the first time that a single cortisol measurement performed two hours after the first morning HC dose strongly correlates with the daytime cortisol profile on a thrice daily regimen, at various HC doses. As stated earlier, a thrice daily regimen has been advocated to avoid supra-physiological plasma cortisol levels [11,25]. and to reduce by two-fold the percentage of daytime single-point cortisol values out of the physiological target range [17]. Herein, we also demonstrate that a single 1000h or 1400h time-point cortisol measurement should predict over-replacement at a cut-off defined in our study conditions which include an HC daily dose splitted into a 50/25/25 percent distribution.

As regards to ACTH, we showed that the predictive value of **Δ**ACTH was less accurate than the measurement of time-point cortisol. Besides, it requires two blood samples drawn at 4-hour interval. Previous studies that have questioned the validity of ACTH plasma level for predicting HC dose adjustment are scarce. Although a low-normal or suppressed morning ACTH concentration has been proposed as a surrogate for HC over-replacement, this criterion has limited value due to the short half-life of hydrocortisone, the uncertainty regarding the normal reference range values and the methodological pitfalls related to ACTH immunoassays at the time these studies were performed [13–16]. Of note, we did not compare the patient’s time-point ACTH concentrations to those from our control group because it was previously shown in patients with Addison’s disease that after HC intake, ACTH concentrations although suppressed remain considerably above the physiological plasma concentrations measured in healthy subjects [16].

Of note, the design of our study did unfortunately not permit to compare the value of the clinical versus biochemical surrogates of HC over-replacement. In a previous study, a clinical and routine biochemical score was compared to time-point cortisol concentrations, and the authors concluded that cortisol time-point measurements could not distinguish over- and under-replacement from adequate replacement [26]. However, the high daily doses used in this study -10 to 14mg/m^2^—probably resulted in a high prevalence of over-replacement clinical symptoms, which may not be observed with current replacement doses. Moreover, time-point cortisol measurements were scarce and drawn randomly after HC intake. Our results, together with the previously reported wide inter-individual variability in the bioavailability of hydrocortisone (17), suggest that a biochemical marker such as time-point cortisol may add information to the sole clinical symptoms for a fine adjustment of HC dose and regimen. The value of plasma cortisol concentration as a predictor of over-replacement is reinforced by our findings that when increasing HC replacement dose, the highest the plasma cortisol AUC is, the strongest the plasma ACTH inhibition is. Our data thus suggest that patients actually are over-replaced based on a surrogate of cortisol over-exposure of tissue targets, *i*.*e*. the glucocorticoid hypothalamic-pituitary feed back on ACTH secretion.

Several limitations of our study might be outlined. One may first emphasize the non-physiological cortisol plasma profile obtained in patients with HC replacement, considering the peaks and troughs of the pharmacokinetic profile. The inter- and intra-individual HC pharmacokinetic variations together with vagaries of HC action at the tissular level may hamper HC replacement efficiency. Many patients are not taking their HC replacement dose using a 50-25-25 splitted daily regimen as done in the present study, moreover patients may vary their HC total dose and daily distribution according to external factors and subjective feeling of their replacement needs. All these pitfalls should be taken into account when judging the actual replacement dose of an individual patient. With all these limitations in mind, our pharmacokinetic algorithm may help adjusting HC dose in a subset of patients with a clearly stable daily replacement regimen in order to determine a baseline dose which should be modified according to daily changing life conditions. Second, the daytime period of cortisol sampling in our study did not include the evening/night and early morning cortisol measurements as performed by Simon *et al*. [17]. These authors have shown that despite a thrice daily regimen including an afternoon HC dose, more than 30% of plasma cortisol concentrations measured at midnight remained out of the physiological target range. Therefore, one may be aware that the current HC regimen used in the present study may exacerbate the tendency to nocturnal hypocortisolism. Third, the high 08.00 am plasma cortisol concentration and its secondary decrease during the 08.00–10.00 am time period observed in healthy volunteers contrasts with the low undetectable baseline plasma cortisol concentration and secondary rise after hydrocortisone intake in patients, resulting in a shift of cortisol AUC between both groups which may hamper the accuracy of their comparative measurements. New formulations of modified-release hydrocortisone tablets may help obtaining a more physiological and stable circadian cortisol profile [27–29].

In conclusion, the present study suggests that current HC doses used for the treatment of Addison’s disease may evoke chronic glucocorticoid over-replacement and that daytime plasma cortisol measurement may help adjusting more physiologically the HC regimen, an important issue considering the consequences of hydrocortisone over-replacement on quality of life, bone remodeling balance and mortality in patients with adrenal deficiency [30, 31, 32]. Our algorithm shows that a single point cortisol determination may be an acceptable surrogate for the evaluation of daytime cortisol AUC and may help the detection of glucocorticoid overexposure during HC replacement. Clinical correlations in real life conditions are still lacking and hydrocortisone dose should still be currently determined on clinical grounds. Further studies may be undertaken for the validation of a pharmacokinetic criteria determining the adequacy of hydrocortisone replacement dose.

## Supporting Information

S1 Supplement Time-point and AUCs for cortisol and ACTH concentrations of AI patients and healthy subjects. (XLS)
